# Clinically unrecognized plasma volume expansion predicts long‐term all‐cause‐mortality in chronic heart failure

**DOI:** 10.1002/clc.23893

**Published:** 2022-08-03

**Authors:** Christoph Ahlgrim, Florian Seiler, Philipp Birkner, Simon Schoechlin, Sebastian Grundmann, Christoph Bode, Torben Pottgiesser

**Affiliations:** ^1^ Department of Cardiology and Angiology II, Heart Center Freiburg University, Faculty of Medicine University of Freiburg Freiburg im Breisgau Germany; ^2^ Department of Cardiology and Angiology I, Heart Center Freiburg University, Faculty of Medicine University of Freiburg Freiburg im Breisgau Germany

**Keywords:** heart failure, mortality, plasma volume

## Abstract

**Introduction:**

Chronic heart failure (CHF) is associated with elevated total blood volume (BV) and distinct phenotypes of total red cell volume (RCV) and plasma volume (PV) elevations. Especially PV expansion during clinical decompensation is linked with adverse clinical outcomes. The role of PV expansion in compensated CHF patients is less clear. Aim of the present study is to investigate the impact of BV parameters on long‐term mortality in CHF patients investigated at a compensated state.

**Methods and Results:**

BV, PV and RCV were determined in 44 (9 female) compensated CHF patients using an abbreviated carbon monoxide method, who were followed up for 6.0 years, (range: 3.7–6.5 years) for all‐cause mortality. In univariate analysis PV expansion but not BV and RCV predicted all‐cause mortality (*p* = .021). A cutoff of 1800 ml PV/m² body‐surface area allows stratification for all‐cause mortality (*p* = .044). PV expansion but not RCV reduction explains the significantly lower hematocrit values of nonsurvivors.

**Discussion:**

In this pilot study, PV expansion, which was unnoticed from a clinician's perspective, but is indicated by significantly lower hematocrit, appears to be a relevant predictor of long‐term all‐cause mortality. Whether PV expansion constitutes an adverse CHF phenotype and can be targeted by diuretic therapy is currently unclear.

## 1 INTRODUCTION

Chronic heart failure (CHF) is frequently associated with an expansion of the total blood volume (BV). In decompensated CHF patients, this elevation of intravascular volume is the key therapeutic target, and a clinically unrecognized increase of the intravascular fluids even after clinical recompensation is associated with increased morbidity and mortality.^1^, ^2^ Even at a clinically compensated state, an increased intravascular volume is frequently observed in CHF patients.^3^, ^4^ This increase in BV is assumed a compensatory mechanism to maintain organ perfusion when cardiac output is decreased. The two constituents of the total blood, plasma volume (PV) and red cell volume^3^, ^4^, ^5^ (RCV) can both be increased in heart failure: most authors have reported an expansion of PV in heart failure patients,^5^, ^6^, ^7^ which predicted adverse outcomes.^1^, ^2^

Elevated values of RCV have also, but not equivocally, been reported in heart failure patients by some authors, including our group reporting on RCV expansion,^3^, ^8^ whereas others did not report on significantly increased RCV expansion in HF patients.^5^ In a recent study, it was shown that CHF patients with an increased RCV have a reduced risk for the occurrence of a composite clinical endpoint of mortality or rehospitalization.^8^ The individual phenotype of the intravascular volumes (e.g. an elevated RCV) appears to be preserved in CHF patients when measured repeatedly over the course of several months.^9^, ^10^

Most work reporting on the impact of directly measured blood compartments on clinical outcomes have emphasized the period directly after discharge after hospital admission.^1^, ^2^, ^11^ Data focusing on the long‐term impact of the volume status is lacking.

Thus, the present study aims to investigate the association of the intravascular volumes with long‐term mortality in a cohort of compensated chronic heart failure patients.

## 2 METHODS

### 2.1 Study design and subjects

During a recruitment visit, BV, RCV, PV, and clinical data were determined in patients with known compensated systolic CHF and a left ventricular ejection fraction <50%, who were referred to our center either for regular follow‐up or specific diagnostic methods such as right‐heart catheterization. The study population has been previously described in more detail.^3^ 47 patients were initially recruited for the study from either the outpatient heart failure clinic or the cardiology ward (if hospitalized for reasons other than cardiac decompensation, e.g. right‐heart catheterization) between June 2014 and July 2015. All subjects provided signed informed consent to participate in the study. Long‐term all‐cause mortality data were retrieved from hospital records, contacting the physician who had initially referred the patient to our hospital, and public records for 44 subjects. Three subjects were lost to follow‐up and were considered missing‐at‐random. The baseline characteristics of the study population are displayed in Table 1. The study design is in line with the latest revised form of the Declaration of Helsinki, the study is approved by the ethics committee of our University Hospital (31/14) and is registered in the German registry for clinical studies (DRKS‐ID: DRKS00006078).

**Table 1:** . Baseline characteristics (*n* = 44)

Parameter	Nonsurvivors (*n* = 9)	Survivors (*n* = 35)	*p*
Sex	6 m/3 f (33%)	29 m/6 f (17%)	.283
Age (years)	59.6 ± 10.1	57.6 ± 8.9	.281
Body‐mass index (kg/m²)	25.2 ± 4.5	28.2 ± 3.5	.034*
NYHA class	2.0 ± 0.7	2.2 ± 0.8	.101
Etiology of heart failure	Ischemic cardiomyopathy 4	Ischemic cardiomyopathy 16	
	Idiopathic cardiomyopathy 4	Idiopathic cardiomyopathy 15	
	Myocarditis 1	Myocarditis 2	
		Hypertensive cardiomyopathy 1	
		Congenital heart disease 1	
Diabetes mellitus (n (%))	0 (0%)	6 (17%)	.181
Blood volume (ml/m² BSA)	3570 ± 646	3294 ± 527	.188
Plasma volume (ml/m² BSA)	2306 ± 473	1989 ± 336	.025*
Red cell volume (ml/m² BSA)	1264 ± 249	1305 ± 228	.633
Hematocrit (%)	39.0 ± 4.3	43.6 ± 3.5	.002*
Serum creatinine (mg/dl)	1.2 ± 0.3	1.1 ± 0.3	.534
proBNP (pg/ml)*	1870 [1468 – 2968]	1030 [458–1601]	.225
LVEF (%)	29 ± 8	30 ± 10	.766
E/e'	18 ± 12	12 ± 5	.102
Vena cava collapse	7 yes/2 no (22%)	25 yes/5 no (20%)***	.712
Sum of diuretics**	1.6 ± 0.9	1.7 ± 0.9	.686

Abbreviations: BNP, B‐type natriuretic peptide; BSA, body surface area; LVEF, Left ventricular ejection fraction.

* Data for proBNP are presented as median ± interquartile range and between‐group differences were compared by the Wilcoxon test due to non‐normality of the distribution of values; 10 values were missing (*n* = 1 in non‐survivors, *n* = 9 in survivors).

** “Sum of diuretics” is the number of individual diuretic substances concomitantly used.

*** For 5 patients, assessment of vena cava was not available.

### 2.2 Endpoint definitions

For the purpose of this study, we evaluated the measurements made at the first determination of intravascular volumes at the recruiting visit. End‐of‐follow‐up (EoF) was December 31, 2020. Patients were either censored at the time of the last contact to our department or at the time of EoF. The primary endpoint was all‐cause death.

### 2.3 Determination of vascular volumes and clinical parameters

BV, RCV, and PV were determined using an abbreviated CO‐rebreathing method,^12^ which has been evaluated in CHF patients before.^13^ In brief, the abbreviated CO‐rebreathing method is a dilutor‐indicator method based on carbon monoxide (CO), which is inhaled by the subject using a closed‐circuit spirometer (SpiCO, Blood Tec) and binds to hemoglobin. By withdrawing capillary samples from the hyperemized earlobe and analyzing these samples for the carboxyhemoglobin level before and after the application of a defined bolus of CO with a standard blood gas analyzer (Radiometer ABL 700series, Radiometer), the total amount of hemoglobin of all erythrocytes (Hb mass) can be precisely determined.^12^ From Hb mass, RCV, PV, and BV can be calculated using simple formulas.^14^

RCV=Hbmass/MCHC×100,

$$\;{\text{BV}\;=\text{RCV}\times 100/\text{hematocrit}}^{*}$$

PV=BV−RCV.

MCHC is the mean corpuscular hemoglobin concentration, and hematocrit is adjusted to the whole body hematocrit using the factor of 0.91 as proposed by Hugh Chaplin Jr. and coworkers.^15^

Clinical data were retrieved from the clinical record of the patient at our hospital. A compensated state was necessary to participate in the study. A physician clinically performed the assessment whether a patient was in a compensated state at the time of inclusion into the study. This assessment was complemented by echocardiographic evaluation of the vena cava (collapsing vs. non‐collapsing).

The echocardiographic assessment was performed by an experienced sonographer at the same day of the CO rebreathing procedure for all outpatients and within the same hospital admission for inpatients using a Philipps IE 33 ultrasound machine (Philipps Healthcare).

### 2.4 Statistical analysis

Data for this study was managed using SAS JMP 9.0; statistical analyses were performed using SPSS. Continuous variables are presented as mean ± SD and categorical variables as frequencies and percentages. A standard t‐test or a chi‐squared test were applied to compare values between groups. For proBNP, non‐parametric statistics (median and interquartile range, Wilcoxon test) were applied due to non‐normal distribution of the values. An area‐under‐the curve‐analysis was used to analyze the predictive value of the plasma volume (PV) (normalized to body surface area, PV_BSA_) for mortality. The Youden‐index, basing on sensitivity and specificity^16^ was chosen to determine an optimal cut‐off value for PV value to predict mortality.

Cumulative event rates were assessed according to the Kaplan–Meier method and compared by a log‐rank test. We derived hazard ratios with associated 95% confidence intervals from Cox proportional hazards models. To adjust for differences in baseline and procedural variables between the strata defined by PV_BSA_ and to identify independent predictors of mortality we fitted uni‐and multivariable Cox models with variables displayed in Table 1 that showed a difference between survivors and non‐survivors after follow‐up at *p* < .1. An alpha level of .05 was accepted for statistical significance.

## 3 RESULTS

The mean follow‐up (of survivors) was 6.0 years, (range: 3.7–6.5 years) in which 9 patients died. The subject characteristics of the surviving patients and the non‐survivors are presented in Table 1.

PV (normalized by body‐surface area, PV_BSA_) was significantly distinct between survivors and non‐survivors. In contrast, the echocardiographic assessment of the vena cava was not different between these groups. The results of the cox regression analysis concerning all‐cause‐mortality as the primary endpoint and relevant predictors are depicted in Table 2. When all significant predictors (PV_BSA,_ and body‐mass‐index) are included in multivariate analysis, PV_BSA_ predicts all‐cause mortality independent from body‐mass‐index.

**Table 2:** Cox regression analysis, all‐cause‐mortality

	Univariate		Multivariate	
	HR (95% CI)	*p*	HR (95% CI)	*p*
PV_BSA_	1.002 (1.000–1.004)	.021*	1.002 (1.000–1.003)	.024
Age	1.026 (0.954–1.103)	.495		
Sex		.200		
Male	Reference			
Female	0.415 (0.103–1.662)	.214		
Body‐mass‐index	0.765 (0.621–0.942)	.015*	0.774 (0.629–0.952)	.015
NYHA class		.354		
III	Reference			
II	3.085 (0.597–15.942)	.701		
I	1.469 (0.207–10.431)	.179		
LVEF	0.995 (0.929–1.065)	.876		
E/e'	1.089 (0.979–1.211)	.116		

Abbreviations: BSA, body surface area; CI, confidence interval; HR, hazard ratio; LVEF, Left ventricular ejection fraction; PV, plasma volume.

No relevant difference was found concerning RCV between survivors and non‐survivors. Thus, the significantly lower hematocrit value observed in non‐survivors is solely due to a PV expansion and not a reduction of RCV in this group. Therefore the hematocrit was excluded from univariate and multivariate modeling.

**Figure 1:**
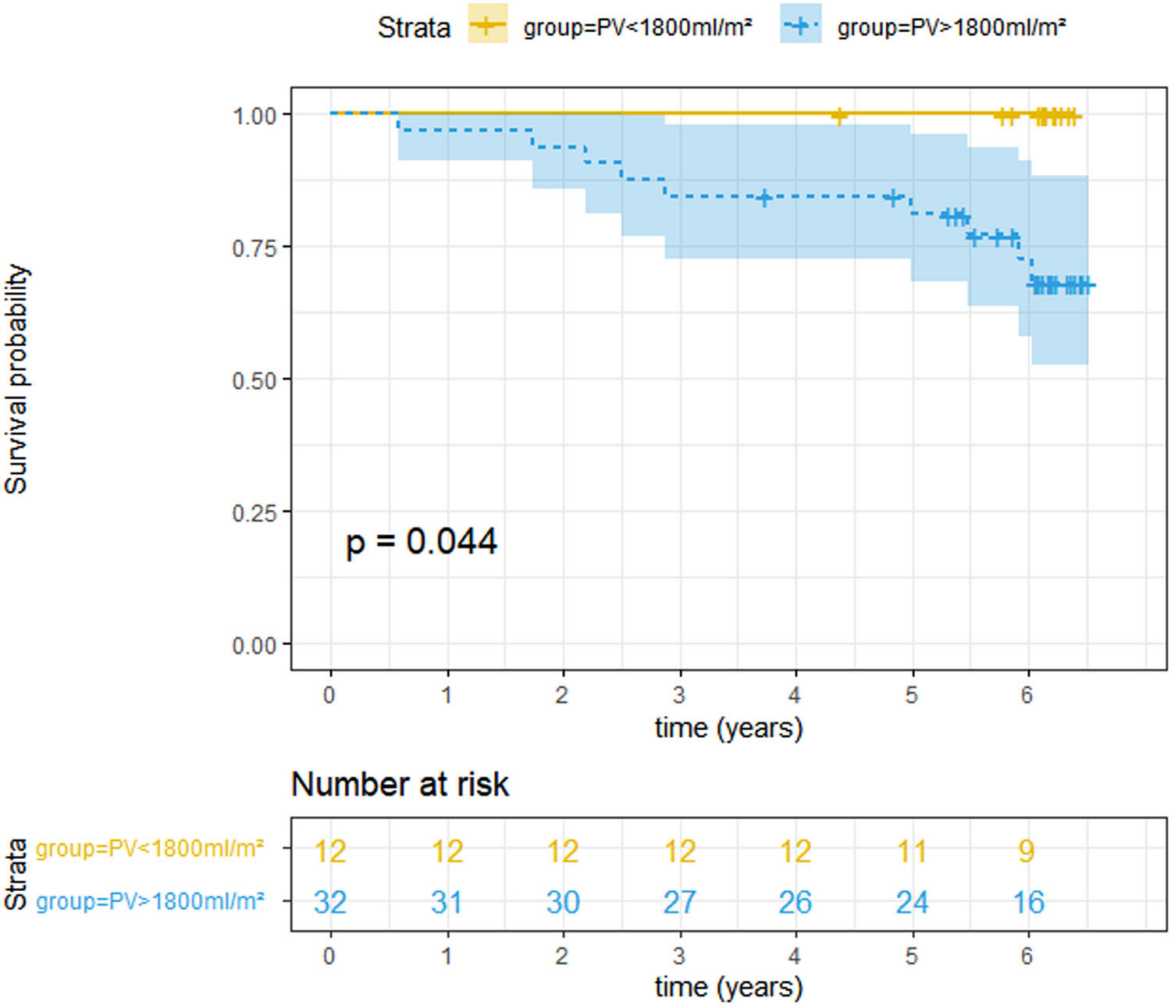
Kaplan–Meyer curve with life‐table displaying survival of the cohort of 44 CHF patients stratified by a PV_BSA_‐cutoff of 1800 ml/m² (shaded area describes 95% confidence intervals for the point estimates).

The area under the curve to predict death within the study course by PV_BSA_ is 0.69 (95% confidence interval 0.49–0.87). The optimal cutoff value for PV_BSA_ to distinguish survivors from non‐survivors estimated by the Youden index is 1800 ml/m². None of the 12 subjects below this threshold died during the observational period, whereas 9 of the 32 subjects above this threshold died. Figure 1 shows the Kaplan–Meyer curve stratified by a PV_BSA_‐cutoff of 1800 ml/m^2^. Table 3 shows the subject characteristics of our cohort when stratified by this PV_BSA_‐cutoff. When stratified by PV_BSA_, the groups differ with respect to all intravascular volumes; in consequence, subjects with PV_BSA_ > 1800 ml/m² feature lower hematocrit values. There is a trend towards an increased number of diuretics in the subjects with a higher PV_BSA_.

**Table 3:** Subject characteristics when stratified by a cutoff value of 1800 ml/m²

Parameter	PV_BSA_ < 1800 ml/m² (*n* = 12)	PV_BSA_ > 1800 ml/m² (*n* = 32)	*p*
Sex	9 m/3 f (25%)	26 m/6 f (19%)	.647
Age (years)	55.9 ± 8.2	58.8 ± 9.4	.351
Body‐mass index (kg/m²)	27.7 ± 2.8	27.5 ± 4.2	.874
NYHA class	2.1 ± 0.8	2.2 ± 0.8	.790
Diabetes mellitus (*n* (%))	1 (8%)	5 (16%)	.530
Blood volume (ml/m² BSA)	2761 ± 285	3572 ± 465	<.001*
Plasma volume (ml/m² BSA)	1641 ± 153	2208 ± 326	<.001*
Red cell volume (ml/m² BSA)	1120 ± 188	1363 ± 210	.001*
Hematocrit (%)	44.4 ± 4.2	42.0 ± 3.9	.074
Serum creatinine (mg/dl)	1.0 ± 0.2	1.2 ± 0.3	.311
proBNP (pg/ml)*	417 [250–1047]	1592 [796–2309]	.007*
LVEF (%)	32 ± 10	29 ± 9	.393
E/e'	12 ± 5	14 ± 8	.418
Sum of diuretics**	1.3 ± 0.6	1.8 ± 1.0	.053

Abbreviations: BSA, body surface area; LVEF, Left ventricular ejection fraction; PV, plasma volume.

* Data for proBNP are presented as median ± interquartile range and between‐group differences were compared by the Wilcoxon test due to non‐normality of the distribution of values.

** “sum of diuretics” is the number of individual diuretic substances concomitantly used.

## 4 DISCUSSION

The major finding of the present study evaluating the prognostic utility of the intravascular volumes in a cohort of CHF patients, who were in a clinically compensated state, is that PV expansion of >1800 ml/m² BSA is associated with a significantly increased mortality risk, whereas no deaths were observed in subjects with a normalized PV below that threshold.

The importance of unrecognized excess of intravascular volumes has been investigated before: In recompensated heart failure patients, unrecognized volume excess predicted rehospitalization and mortality during a median follow‐up of approximately 2‐years^1^ and 180 days.^2^ To the best of our knowledge, the follow‐up of more than 2100 days of our study presents the longest follow‐up of a cohort of patients for whom the intravascular parameters were determined at a compensated state. Remarkably, mortality in our cohort was not solely determined by early deaths within the first years of observation, but also by deaths occurring after the third year of follow‐up. This might point towards the distinct importance of early detection (and possibly treatment) of PV expansion in CHF patients. In this context, guiding decongestion by establishing intravascular volumes has been shown to be associated with better outcomes in CHF patients in a propensity‐score‐matched control design.^11^ Unconditional use of diuretics, especially loop diuretics, is not encouraged by current guidelines.^17^ Diuretics should only be used to reduce signs and symptoms of congestion.^17^ A clear effect of diuretics use on mortality has not been established, however, diuretics use has been associated with a reduction of the risk of worsening disease and improved exercise capacity.^18^ In this context, proBNP, which was not included in the uni‐ or multivariate analysis because no significant difference was observed between survivors and non‐survivors, is significantly increased in subjects with a PV > 1800 ml/m², who also feature significantly higher RCV and BV. A decongestion strategy guided by BNP has been shown to improve survival in CHF.^19^ Our data, therefore, highlights the importance of recognition of proBNP, which, also in our cohort, is associated with an excess of intravascular volumes and, most probably consequential, survival.

When determining vascular volumes in CHF patients, distinct types of alterations of PV and RCV occur in non‐anemic subjects. First, some subjects feature a PV expansion but not an RCV expansion leading to lower hematocrit values without anemia.^5^ A second group of CHF patients features an RCV expansion without PV expansion, and a third group featuring expansions of both compartments.^3^, ^7^ Recent data has highlighted a protective role of an elevated RCV and the consequent polycythemia in CHF patients.^2^ The alterations of intravascular volumes appear to be preserved over time,^9^, ^10^ whilst the mechanisms by which these alterations are mediated remain unclear. Remarkably, when compared with a CHF cohort, previous CHF patients after heart transplantation feature the phenotype of a healthy cohort and do not show significant expansion of the PV or RCV.^20^

Importantly, an expansion of BV does not necessarily translate into an elevated filling pressure of the left ventricle in CHF, because these two factors are only moderately correlated.^21^ Just recently, and aiming to understand the role of the complex interaction between BV and intracardiac pressures in cardiac congestion better, Dmitry Yaranov and coworkers highlighted the necessity to specifically consider the volume distribution to the central vascular compartment and the venous tone rather than whole intravascular compartment reflected by BV or PV alone.^22^

In our cohort, RCV and BV did not predict outcomes and were not different between survivors and non‐survivors. Therefore, PV expansion was associated with significantly lower hematocrit values in non‐survivors pointing towards a key importance of this universally available parameter when planning diuretic therapy. However, when comparing diuretics use between those subjects with an elevated PV (Table 3), the number of diuretics is higher (with a nonsignificant trend) in those subjects with an elevated PV (with PV_BSA_ > 1800 ml/m^2^). Whether this is due to the history of the patients, possibly featuring previous decompensations at the time of inclusion into the study, or there is a link between an impaired kidney function due to diuretics use, consequently leading to an increased PV, cannot be clarified by our study. Nevertheless, subclinical congestion is a frequent observation in heart failure patients.^23^

Investigation of intravascular volumes has a long history in CHF.^6^, ^24^, ^25^ Our cohort can be considered a contemporary cohort, because treatment with mineralocorticoid receptor antagonists^26^, ^27^ and angiotensin receptor‐neprilysin inhibitors^28^ was available during the study course, although no patient was treated with angiotensin‐receptor‐neprilysin inhibitors at initiation of the study; as it was only recommended by major heart failure guidelines in 2016.^29^ Inhibitors of sodium‐glucose Cotransporter‐2 (SGLT2) were not used at the time of inclusion into the study, and most probably were not used widely during most of the later course of our study. SGLT‐2 inhibitors have the specific feature of lowering the circulating PV,^30^ which, hypothesizing, may be one of the main features of lowering mortality in HFrEF patients. SGLT‐2‐inhibitors are currently recommended as a standard treatment for heart failure.^17^ Our work, featuring 32/44 (72%) of CHF patients with a PV_BSA_ of >1800ml/m² thus indirectly supports the importance of early initiation of therapy with an SGLT‐2‐inhibitor at an early stage.

## 5 LIMITATIONS

First, the prospective but observational design of this study does not allow a cause‐and‐effect relationship to be established. Second, even when analyzing the present data with care, there is the inherent limitation of possible bias due to unmeasured confounding factors: All patients were recruited at a single center, which might lead to selection bias. Women were underrepresented in the study sample; however, sex did not appear as a relevant predictor for mortality in our cohort. Third, by design, we cannot elucidate the mechanism(s) behind the observation of some HF patients developing PV expansion while others do not. This is a key issue in further understanding the pathophysiology of chronic HF.

Finally, by design of the study, we assessed all‐cause mortality only. Therefore, we cannot distinguish between cardiovascular and non‐cardiovascular causes of death, but probably underestimate the true association of PV excess with cardiovascular mortality.

## 6 CLINICAL IMPLICATION

Despite the safety and general feasibility of the method, the clinical applicability of establishing the vascular parameters by CO rebreathing is limited by a somewhat lengthy experiment and the restriction of patients to NYHA levels below IV. However, our work contributes to understanding the impact of the pathophysiological expansion of PV. The significantly decreased hematocrit values observed in non‐survivors are explained by PV expansion but not RCV reduction. Therefore, from a clinician's point of view, it appears important to assess the individual course of hematocrit values to detect early signs of PV excess (and possibly, “subclinical decompensation”) when hematocrit values are decreasing. In this context estimating PV or other blood volumes from hematocrit and sex^31^ may be of a limited value for as they are only moderately associated with true PV excess.^32^ Moreover, our data emphasizes on recognition of proBNP as an indicator of excess intravascular volumes in stable CHF.

## AUTHOR CONTRIBUTIONS

Christoph Ahlgrim contributed to conception and design, analysis and interpretation of data, drafting and revising the manuscript. Torben Pottgiesser contributed to conception and design, interpretation of the data, and drafting and revising the manuscript. Philipp Birkner and Florian Seiler contributed to acquisition of data as well as interpretation of data, and revising the manuscript. Simon Schoechlin, Sebastian Grundmann, and Christoph Bode contributed to conception and design, and revising the manuscript critically for important intellectual content. All above authors have approved the final manuscript.

## CONFLICTS OF INTEREST

The authors declare no conflicts of interest.

## Data Availability

Data available on reasonable request from the authors.

## References

[1] Androne AS , Hryniewicz K , Hudaihed A , Mancini D , Lamanca J , Katz SD . Relation of unrecognized hypervolemia in chronic heart failure to clinical status, hemodynamics, and patient outcomes. Am J Cardiol. 2004;93:1254‐1259. 15135699 10.1016/j.amjcard.2004.01.070

[2] Kelly KL , Wentz RJ , Johnson BD , Miller WL . Relation of intravascular volume profiles to heart failure progression and clinical outcomes. Am J Cardiol. 2021;153:65‐70. 34215355 10.1016/j.amjcard.2021.05.020 PMC8316305

[3] Ahlgrim C , Birkner P , Seiler F , et al. Increased red cell volume is a relevant contributing factor to an expanded blood volume in compensated systolic chronic heart failure. J Card Fail. 2020;26:420‐428. 31790816 10.1016/j.cardfail.2019.11.025

[4] Miller WL . Fluid volume overload and congestion in heart failure: time to reconsider pathophysiology and how volume is assessed. Circ Heart Fail. 2016;9:e002922. 27436837 10.1161/CIRCHEARTFAILURE.115.002922

[5] Adlbrecht C , Kommata S , Hülsmann M , et al. Chronic heart failure leads to an expanded plasma volume and pseudoanaemia, but does not lead to a reduction in the body's red cell volume. Eur Heart J. 2008;29:2343‐2350. 18701467 10.1093/eurheartj/ehn359

[6] Gunton RW , Paul W . Blood volume in congestive heart failure. J Clin Invest. 1955;34:879‐886. 14381518 10.1172/JCI103144 PMC1072619

[7] Miller WL , Mullan BP . Volume overload profiles in patients with preserved and reduced ejection fraction chronic heart failure: are there differences? A pilot study. JACC Heart Fail. 2016;4:453‐459. 26970830 10.1016/j.jchf.2016.01.005

[8] Miller WL , Strobeck JE , Grill DE , Mullan BP . Blood volume expansion, normovolemia and clinical outcomes in chronic human heart failure‐more is better. Am J Physiol‐Heart Circ Physiol . 2021. 10.1152/ajpheart.00336.2021 PMC9095049 34676782

[9] Ahlgrim C , Seiler F , Birkner P , et al. Time course of red cell volume and plasma volume over six months in compensated chronic heart failure. *ESC Heart Fail* [Internet]. 2021. https://onlinelibrary.wiley.com/doi/abs/10.1002/ehf2.13179 10.1002/ehf2.13179 PMC8006671 33403801

[10] Miller WL , Albers DP , Gansen DN , Mullan BP . Intravascular volume profiles in patients with class I and II systolic heart failure: heterogeneity and volume overload are common even in mild heart failure. J Card Fail. 2018;24:417‐424. 28982634 10.1016/j.cardfail.2017.09.010

[11] Strobeck JE , Feldschuh J , Miller WL . Heart failure outcomes with volume‐guided management. JACC Heart Fail. 2018;6:940‐948. 30316941 10.1016/j.jchf.2018.06.017

[12] Schmidt W , Prommer N . The optimised CO‐rebreathing method: a new tool to determine total haemoglobin mass routinely. Eur J Appl Physiol. 2005;95:486‐495. 16222540 10.1007/s00421-005-0050-3

[13] Ahlgrim C , Birkner P , Seiler F , et al. Applying the optimized CO rebreathing method for measuring blood volumes and hemoglobin mass in heart failure patients. *Front Physiol*. 2018. https://www.frontiersin.org/articles/10.3389/fphys.2018.01603/full 10.3389/fphys.2018.01603 PMC6240604 30483155

[14] Heinicke K , Wolfarth B , Winchenbach P , et al. Blood volume and hemoglobin mass in elite athletes of different disciplines. Int J Sports Med. 2001;22:504‐512. 11590477 10.1055/s-2001-17613

[15] Chaplin H , Mollison PL , Vetter H . The body/venous hematocrit ratio: its constancy over a wide hematocrit range. J Clin Invest. 1953;32:1309‐1316. 13108998 10.1172/JCI102859 PMC438476

[16] Youden WJ . Index for rating diagnostic tests. Cancer. 1950;3:32‐35. 15405679 10.1002/1097-0142(1950)3:1<32::aid-cncr2820030106>3.0.co;2-3

[17] McDonagh TA , Metra M , Adamo M , et al. ESC guidelines for the diagnosis and treatment of acute and chronic heart failure: developed by the task force for the diagnosis and treatment of acute and chronic heart failure of the European Society of Cardiology (ESC) with the special contribution of the Heart Failure Association (HFA) of the ESC. Eur Heart J. 2021;2021(42):3599‐3726.

[18] Faris R , Flather M , Purcell H , Henein M , Poole‐Wilson P , Coats A . Current evidence supporting the role of diuretics in heart failure: a meta analysis of randomised controlled trials. Int J Cardiol. 2002;82:149‐158. 11853901 10.1016/s0167-5273(01)00600-3

[19] Pfisterer M , Buser P , Rickli H , et al. TIME‐CHF investigators. BNP‐guided vs symptom‐guided heart failure therapy: the trial of intensified vs standard medical therapy in elderly patients with congestive heart failure (TIME‐CHF) randomized trial. JAMA. 2009;301:383‐392. 19176440 10.1001/jama.2009.2

[20] Seiler F , Ahlgrim C , Birkner P , et al. Blood volume and hemoglobin mass in long‐term heart transplant recipients with and without anemia. J Cardiothorac Surg. 2021;16:1‐12. 34078389 10.1186/s13019-021-01510-1 PMC8171054

[21] Miller WL , Sorimachi H , Grill DE , Fischer K , Borlaug BA . Contributions of cardiac dysfunction and volume status to central haemodynamics in chronic heart failure. Eur J Heart Fail. 2021;23:1097‐1105. 33565251 10.1002/ejhf.2121 PMC8665273

[22] Yaranov DM , Jefferies JL , Silver MA , Burkhoff D , Rao VN , Fudim M . Discordance of pressure and volume: potential implications for pressure‐guided remote monitoring in heart failure. J Card Fail. 2022;28:870‐872. 35158025 10.1016/j.cardfail.2022.02.003 PMC9804116

[23] Cuthbert JJ , Pellicori P , Flockton R , et al. The prevalence and clinical associations of ultrasound measures of congestion in patients at risk of developing heart failure. Eur J Heart Fail. 2021;23:1831‐1840. 34632680 10.1002/ejhf.2353

[24] Gibson JG , Evans WA . Clinical studies of the blood volume. III. Changes in blood volume, venous pressure and blood velocity rate in chronic congestive heart failure. J Clin Invest. 1937;16(16):851‐858. 16694531 10.1172/JCI100911 PMC424924

[25] Seymour WB , Pritchard WH , Longley LP , Hayman JM . Cardiac output, blood and interstitial fluid volumes, total circulating serum protein, and kidney function during cardiac failure and after improvement. J Clin Invest. 1942;21:229‐240. 16694906 10.1172/JCI101294 PMC435134

[26] Pitt B , Zannad F , Remme WJ , et al. The effect of spironolactone on morbidity and mortality in patients with severe heart failure. N Engl J Med. 1999;341:709‐717. 10471456 10.1056/NEJM199909023411001

[27] Pitt B , Remme W , Zannad F , et al. Eplerenone Post‐Acute myocardial infarction heart failure efficacy and survival study investigators. eplerenone, a selective aldosterone blocker, in patients with left ventricular dysfunction after myocardial infarction. N Engl J Med. 2003;348:1309‐1321. 12668699 10.1056/NEJMoa030207

[28] McMurray JJ , Packer M , Desai AS , et al. Angiotensin–neprilysin inhibition versus enalapril in heart failure. N Engl J Med. 2014;371:993‐1004. 25176015 10.1056/NEJMoa1409077

[29] Ponikowski P , Voors AA , Anker SD , et al. ESC guidelines for the diagnosis and treatment of acute and chronic heart failure: the task force for the diagnosis and treatment of acute and chronic heart failure of the European Society of Cardiology (ESC). Developed with the special contribution of the Heart Failure Association (HFA) of the ESC. Eur J Heart Fail. 2016;2016(18):891‐975. 10.1002/ejhf.592 27207191

[30] Heerspink HJ , De Zeeuw D , Leslie B , List J . Dapagliflozin a glucose‐regulating drug with diuretic properties in subjects with type 2 diabetes. Diabetes Obes Metab. 2013;15:853‐862. 23668478 10.1111/dom.12127 PMC3906841

[31] Ling HZ , Flint J , Damgaard M , et al. Calculated plasma volume status and prognosis in chronic heart failure. Eur J Heart Fail. 2015;17:35‐43. 25469484 10.1002/ejhf.193

[32] Fudim M , Miller WL . Calculated estimates of plasma volume in patients with chronic heart failure–comparison to measured volumes. J Card Fail. 2018;24:553‐560. 30098381 10.1016/j.cardfail.2018.07.462 PMC6196104

